# Repurposing a rare opportunity: a brief insight into how implicit bias towards biomedicine impacts the care received by patients with a rare illness

**DOI:** 10.1186/s13023-019-1024-6

**Published:** 2019-02-28

**Authors:** Logan Zane John Williams

**Affiliations:** Faculty of Medical and Health Sciences, The University of Auckland, 85 Park Road, Grafton, Auckland, 1023 New Zealand

**Keywords:** Rare illness, Medical student, Implicit bias, Psychosocial care

## Abstract

Medical students automatically couple rare illnesses with biomedical minutiae. Upon meeting CS (pseudonym), a 5-year-old boy with Worster Drought Syndrome, I became inadvertently caught in the trap of focusing on his diagnosis rather than CS as a patient. I fumbled around CS’s past medical history by fervently asking about all the different types of seizures he was plagued by. It was only after CS’s mother, TS (pseudonym), volunteered the psychosocial challenges she faced caring for CS that I realised the strong implicit bias I had towards biomedical aspects of patient care. I discovered that TS was robbed of being able to celebrate CS’s developmental milestones, as they posed unique challenges with very serious risks. Having learned the extent of such psychosocial challenges, I searched to understand the origin of biases towards biomedicine, within myself and within the medical system. I attribute my own biases in part to the current state of medical education, which disproportionately focuses on the scientific, rather than psychosocial and humanistic factors. Systemically, there is a large commercial driving force behind scientific research into rare illnesses. The interest in rare illnesses displayed by pharmaceutical industries only after the incentivisation by various countries highlight the socio-political constraints that bind research in this field. These biases, along with the marginalisation of patients and families with rare illnesses, means there is a very real risk that the goals of all stakeholders are incongruous. As such, it is imperative that we give these patients and families a voice.

## Main text

I met CS, a 5-year-old boy, and his mother TS in the emergency department of a rural hospital. He presented because TS had noticed a change in his seizure pattern and frequency after a fall. It was discovered that the offending pathology was a subdural haematoma that eventually required neurosurgery. Yet, the investigative work of the paediatric team was made more challenging because of CS’s complicated medical history that included Worster-Drought Syndrome (WDS).

Before meeting CS, I had no understanding of what WDS was. CS’s story wasn’t a common illness script that a medical student would hear about. Despite being briefed on CS by one of the foundation year doctors, I fumbled around CS’s past medical history by fervently asking about all the different types of seizures he was plagued by. I was determined to solve this complex biomedical puzzle. It was only after TS volunteered the psychosocial challenges she faced whilst caring for CS that I realised the strong implicit bias I had towards the biomedical aspects of patient care. A striking example of such challenges was the risk CS faced as he became stronger and increasingly independent. For many parents, their children achieving developmental milestones like walking are celebrated. But for TS, this meant that CS was able to climb over the railing of his bed. This newly developed mobility, combined with his seizures, posed a very serious risk of head injury. Without protective headwear, TS attempted to mitigate this risk by rearranging the entire family home. With TS, CS now sleeps on mattresses in the lounge, which contains almost no furniture. Undoubtedly, such information about the home environment would’ve had significant bearing on the synthesis of a management plan.

What if TS had not been so forthcoming with this information? Getting inadvertently caught in the trap of focusing on the disease rather than the person can result in neglecting general treatment and preventive care [1]. Sadly, the term ‘treatment’ for a medical student evokes reflexive thoughts about pharmacotherapy, leaving psychosocial and humanistic aspects as a mere afterthought. Yet, addressing the non-biomedical aspects of living are equally if not more important, because they are also amenable to intervention, and are just as likely to make a long-lasting and significant impact. Since the harmful consequences of CS’s seizures became evident, TS had been involved in an ongoing saga to acquire government funded protective headwear. Had CS received the headwear in a timely fashion, he might not have required this current hospital admission or neurosurgery. This terrible circumstance underscores the considerable effect of psychosocial factors on health outcomes, and how the physical and social environment can strongly shape the course of an illness [2]. Although we will never know the true impact of this subdural haematoma on CS’s future, one cannot help but ponder the what ifs.

Acknowledging our personal implicit bias towards biomedicine is good professional practice, but it would be negligent not to delve further and question its origins. Using future doctors as an example, one culprit may be the structure and content of our current medical education. There are still undertones of outdated, paternalistic and short-sighted models of conceptualising illness. One such model is the ‘disease’-based approach to medicine, where ‘disease’ is defined as a purely pathophysiologic process [2]. The result is a disproportionate focus on scientific underpinnings in medical school, which are positively reinforced when medical students are rewarded for being able to recall biomedical minutiae rather that displaying the ability to think critically and holistically. Regrettably, this model disregards the psychosocial and humanistic aspects of patient care, but there are certainly models that we can adopt to improve the care of patients with rare illnesses. Having highlighted the deleterious consequences that can occur when such psychosocial and humanistic factors are neglected, we should endeavour to take an illness-based approach, which encompasses physical, psychosocial, and cultural factors [2].

This disease-based approach also assumes that patients and families have a poorer understanding of their condition compared to the medical profession. An fundamental element of this model is the belief held by the medical profession that patient understanding must be underpinned by extensive knowledge of the aetiology and pathophysiology of their illness. Perhaps we should admit that a theoretical knowledge of an illness, along with a brief snapshot of their life in a clinical setting, is not a substitute for a lifetime worth of experience. Over this lifetime they’ve become experts, and managing the unique challenges of their illness outside this clinical setting is second-nature. It is a humbling realisation that it was actually TS who knew the intricacies of CS’s illness, and without her guidance, the paediatric team would have been stumped.

After hearing about CS’s journey, I developed a greater appreciation for the psychosocial impact living with WDS had on him and his family. Having now realised the extent of my own biases, these same biases became apparent throughout the medical system. For instance, research efforts into rare illnesses are enrapt with biomedicine. Prior to 1983, rare illnesses were neglected by the medical system. These ‘health orphans’ were a challenge for the medical community for a number of reasons. The National Commission on Orphan Disease highlighted issues that affected patients’ care, such as little information on rare illnesses, difficulties of financing research, and the limited availability of effective treatments [3]. In order to stimulate interest in patients with rare illnesses, several countries passed legislation and implemented directives that incentivised pharmaceutical companies to develop treatments for patients with rare illnesses, such as market exclusivity, tax credits and accelerated marketing procedures [4].

Since the introduction of such legislations, the pharmaceutical industry has become increasingly interested in finding therapeutics for rare illnesses. The market for ‘orphan drugs’ was estimated to be worth over USD $80 billion in 2009, in part because of the governmental incentivisation, but also because of high drug prices and relatively low competition [5]. There is also a greater appreciation of the potential market size, as although rare illnesses are individually rare, they are surprisingly common as a collective [6]. Yet, we don’t actually know if this is the most effective approach to rare diseases, or if it would be more responsible to allocate funding towards psychosocial and humanistic factors. Would research into rare illnesses still receive the same amount of attention if the financial benefits for the pharmaceutical industry weren’t so obvious? This is not to say that the pharmaceutical industry isn’t also invested in helping patients with rare illnesses. It would be fiscally irresponsible for any business to pursue a venture that didn’t guarantee at least some return. Rather, it highlights the economic and socio-political constraints that this scientific field is bound by. We must do away with our biomedical biases, and determine if research into more holistic aspects of rare illnesses is warranted. If this is the case, then such research needs to be made financially viable. The exemplar of the incentivisation of drug development and discovery proves that socio-political organisations have the means to bring about such change.

Given all of this we must ask ourselves, “who is making the decisions about the future of patients with rare illnesses?” I was fortunate enough to hear about the grit and perseverance that fuelled TS and the rest of the family in caring for CS. It seemed that TS in particular had sacrificed every aspect of her life in this pursuit. “But I wouldn’t change a thing,” she told me. Since having CS, TS had developed a greater appreciation for aspects of life that many of us take for granted every day. Of course, this is only the opinion of one carer for a patient with a rare illness, and TS’s stance is not generalisable to others with rare illnesses. Admittedly, accepting and loving a child for who they are, and also wanting a cure for their rare illness are not mutually exclusive. The point is that the desires of patient and family (whether it be cure, or provision of tools to allow better participation in society) should be the decision of the patient and their family. Furthermore, it is imperative that the goals of medical and socio-political stakeholders are aligned. How can we achieve unison between all stakeholders when patients and families with rare illnesses are so marginalised? We must start by giving these patients and families a platform so that they are heard by all relevant stakeholders.

Being able to voice the journey of CS and TS through my perspective has been a very special opportunity. Medical students automatically couple rare illnesses with biomedical minutiae, and it would have been all too easy hone in on CS’s diagnosis of WDS. If it were not for TS, I would’ve denied myself the rare opportunity to think critically about wider aspects of patient care and the factors that influence them. TS’s recount of CS’s journey highlights the dangers of losing sight of the bigger picture, of treating the disease rather than the illness. I hope that other future doctors actively pursue, and learn from, the CS’s and TS’s of this world.

## Abbreviation

WDS: Worster-Drought Syndrome
